# Older adults’ and caregivers’ perceptions about urinary tract infection and asymptomatic bacteriuria guidelines: a qualitative exploration

**DOI:** 10.1017/ash.2023.498

**Published:** 2023-12-04

**Authors:** Michael J. Durkin, Viktoria Schmitz, Kevin Hsueh, Zoe Troubh, Mary C. Politi

**Affiliations:** 1 Division of Infectious Diseases, Department of Internal Medicine, Washington University School of Medicine, St. Louis, MO, USA; 2 Division of Public Health Sciences, Department of Surgery, Washington University School of Medicine, St. Louis, MO, USA

## Abstract

**Objective:**

To explore older adults’ and caregivers’ knowledge and perceptions of guidelines for appropriate antibiotics use for bacteria in the urine.

**Design:**

Semi-structured qualitative interviews.

**Setting:**

Infectious disease clinics, community senior living facilities, memory care clinics, and general public.

**Participants:**

Patients 65 years or older diagnosed with a urinary tract infection (UTI) in the past two years, or caregivers of such patients.

**Methods:**

We conducted interviews between March and July 2023. We developed an interview guide based on the COM-B (capability, opportunity, motivation-behavior) behavior change framework. We thematically analyzed written transcripts of audio-recorded interviews using inductive and deductive coding techniques.

**Results:**

Thirty participants (21 patients, 9 caregivers) enrolled. Most participants understood UTI symptoms such as pain during urination and frequent urination. However, communication with multiple clinicians, misinformation, and unclear symptoms that overlapped with other health issues clouded their understanding of asymptomatic bacteriuria (ASB) and UTIs. Some participants worried that clinicians would be dismissive of symptoms if they suggested a diagnosis of ASB without prescribing antibiotics. Many participants felt that the benefits of taking antibiotics for ASB outweighed harms, though some mentioned fears of personal antibiotic resistance if taking unnecessary antibiotics. No participants mentioned the public health impact of potential antibiotic resistance. Most participants trusted information from clinicians over brochures or websites but wanted to review information after clinical conversations.

**Conclusion:**

Clinician-focused interventions to reduce antibiotic use for ASB should also address patient concerns during clinical visits, and provide standardized high-quality educational materials at the end of the visit.

## Background

Asymptomatic bacteriuria (ASB) is a benign condition in which a patient has bacteria in their urine without symptoms of a urinary tract infection (UTI).^1^ Asymptomatic bacteriuria is often misdiagnosed by clinicians and patients as a UTI following over-interpretation of positive urine cultures.^2^ This is common among older adults^3,4^ who are at the highest risk for significant side effects from unnecessary antibiotics.^5,6^ Inappropriate antibiotic use for ASB leads to adverse events^7^ and contributes to global antibiotic resistance.^2,8^ Experts estimate that 10 million people could die annually due to antibiotic resistance globally by 2050.^9^

Clinical decision support can reduce overuse of antibiotics for ASB.^10^ However, to our knowledge, no decision tools for ASB have incorporated patients’ and caregivers’ perspectives. Without these perspectives, clinicians and patients may have different expectations about UTI or ASB management. For example, patients and caregivers often report confusion about true UTI symptoms, particularly among older patients, as age-related changes are often hard to differentiate from symptoms of a UTI.^11^ Patients and caregivers often worry that malodorous urine, cloudy urine, isolated confusion, or behavior changes are UTI symptoms and seek antibiotics for treatment; these views conflict with clinical guidelines.^12–14^

Patient satisfaction is important to clinicians considering prescribing antibiotics. Even with education and decision support, clinicians can be hesitant to follow antibiotic guidelines if they fear lower patient satisfaction or an impact on the patient-clinician relationship.^15–18^ Patient and caregiver engagement is essential to adapt the content, format, and process of delivering interventions de-implementing inappropriate antibiotic prescribing for ASB. We explored patients’ and caregivers’ knowledge and perceptions of guidelines for treating bacteria in the urine. We asked how information could support antibiotics discussions and clinical or public health messaging. We also asked about resources patients and caregivers might want to help them understand UTI and ASB management.

## Methods

The Washington University Human Research Protection Office approved this project as exempt research (HRPO# 202211197). Eligibility criteria included patients aged 65 years or older who were diagnosed with a UTI in the past two years or caregivers of such patients. We recruited patients through flyers, online advertisements, newsletters, memory care clinics, medical clinics, and senior living centers. Potential participants contacted the research team to participate. Participants could also invite others to participate (snowball sampling^19^).

Eligible and interested participants provided informed consent prior to interviews. A study staff member trained in qualitative interviewing, supervised by the principal investigator, conducted all interviews. Interviews occurred via telephone, teleconference technology, or in person based on participants’ preferences. Interviews were audio-recorded with consent.

**Figure 1.**
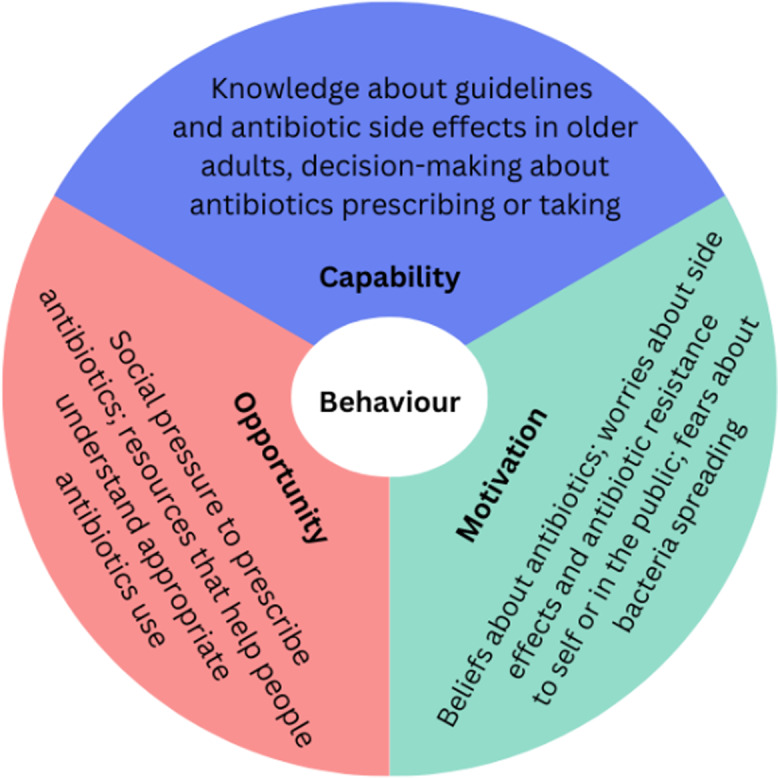
Conceptual model of behavior change guiding interviews and analyses, adapted from the behavior change wheel.^21^

A semi-structured qualitative interview guide was created based on a behavior change framework involving capability, opportunity, and motivation to engage in behaviors (Figure 1).^20^ The interview guide asked questions related to participants’ experiences, thoughts, and feelings regarding treatment for UTIs and ASB. Participants were also asked about strategies that would help older adults and caregivers understand UTI and ASB management. They were shown an example evidence-based resource and were asked for reactions to it. Appendix A shows the interview guide used during the interview.

Participants received a $30 gift card for completing the interview. Audio recordings were transcribed verbatim. A codebook was developed and refined based on the theoretical framework and emerging themes. Transcripts were coded by one of two trained coders (VS, ZT) using inductive coding. Using the constant comparative method, each coder separately coded the transcripts and compared results until inter-rater reliability was reached (kappa >0.75 and >95% agreement on each code) which occurred after the first five transcripts. The remaining 25 transcripts were coded separately by one of the raters. Coders discussed any questions and a third team member (MP) was consulted if consensus was not reached.

## Results

Thirty participants enrolled out of 39 approached and eligible (77% response rate). Five potential participants were unable to be reached, and four declined due to lack of time or interest. Table 1 displays demographic characteristics of enrolled participants. Enrolled participants were mostly female (93%) and white (83%), with at least some college education.

**Table 1.** Participant characteristics

	Patients (*N* = 21)	Caregivers (*N* = 9)	All (*N* = 30)
**Age**	Mean = 77.6 (SD 7.4, range 31–70)	Mean = 57.2 (SD 10.8, range 65–89)	Mean = 71.5 (SD 12.7, range = 34–89)
**Gender Identity of Participants**			
Female	21 (100%)	9 (100%)	28 (93%)
Male	0 (0%)	0 (0%)*	2 (7%)
**Race**			
Black or African American	3 (14%)	1 (11%)	4 (13%)
White	18 (86%)	7 (78%)	25 (83%)
Another Race	–	1 (11%)	1 (3%)
**Highest level of formal education**			
HS diploma, GED or less	2 (10%)	1 (11%)	3 (10%)
Some college	5 (24%)	–	5 (17%)
College degree or more	14 (67%)	8 (89%)	22 (73%)
**Household income last year**			
Less than $30,000	3 (14%)	0 (0%)	3 (10%)
At least $30,000, <$60,000	2 (10%)	4 (44%)	6 (20%)
At least $60,000, <$75,000	5 (24%)	0 (0%)	5 (17%)
$75,000 or more	4 (19%)	3 (33%)	7 (23%)
Prefer not to answer	7 (33%)	2 (22%)	9 (30%)

*Note*. *Although no participants were male, two caregivers described experiences of male patients. Note that only patients aged 65 or older or their adult caregivers were allowed to participate in the study.

Several themes emerged from the participant interviews, which we categorized according to our conceptual framework displayed in Figure 1.

**Theme 1 (Capability): Most older adults and caregivers understood symptoms of UTIs such as burning during urination, frequent urination, pain, or new onset urinary urgency.** For example, when asked about symptoms of a recently diagnosed UTI, participants mentioned:

*“Frequency, urgency, and burning. Feeling like you have to go even when you don’t.” (Participant 001, patient)*

*“The mainstream symptom is frequency of urination…it’s like my plumbing never stops…when this frequency starts, and then when you go…sometimes there’s pain.” (Participant 015, patient)*

*“When I urinate, it hurts. I had pain when it actually came out…I have urgency all the time…I keep my eye on the bathroom so I can find it.” (Participant 021, Patient)*

Caregivers also understood changes in symptoms that prompted seeking care for their loved one’s potential UTI:

*“She had burning when she urinated…She does have a frequent urge to urinate.” (Participant 22, caregiver)*

**Subtheme 1a:** However, many symptoms were confusing to older adults and their caregivers because they are hard to differentiate from other ageing-related changes. As one participant noted:

*“The frequency is getting more frequent as I get older, so it’s hard to tell if the frequency is normal frequency, or urinary tract infection frequency.” (Participant 23, Patient)*

Caregivers agreed that UTI symptoms were not always as clear for older adults who suffer from dementia or mild cognitive impairment:

*“UTIs…it does seem to have an impact on her on her mental status… When she’s had the UTIs, those stories get more complex. She gets more agitated…I don’t think all medical professionals understand or believe that bacteria in the urine can have symptoms that are cognitive or mental in nature.” (Participant 024, Caregiver)*

*“I know that sometimes it [UTIs] can affect elderly more harshly than it does younger people…they can become a little senile and have some memory loss.” (Participant 017, Caregiver)*

**Theme 2 (Opportunity): Communication with multiple clinicians and misinformation can make diagnosing and treating UTIs challenging.**

Participants found communicating with multiple clinicians about symptoms challenging. One caregiver remarked on the burden of repeating information to different treating clinicians who might respond differently to symptoms:

*“You’re never dealing with the same nurse. I know all those nurses, but they’re not there every day….The communication is challenging in a nursing home because of the different people that you see every day…It’s a little slower getting the ball rolling to get him to the next steps. They notice that he has symptoms. The nurse has to go to the doctor. The doctor has to, I’m sure, put the order in. Then they have to wait for the test.” (Participant 008, Caregiver)*

One patient commented that her doctor and a nurse gave different information about whether treatment was necessary:

*“When I was in the hospital [for a heart condition]…the nurse thought it was [a UTI], but the doctor…refuted that… they test everything, if you’re in the hospital for anything…[but with no symptoms of a UTI], I think I was just relieved [that I didn’t need antibiotics] because I thought, “Good, I won’t have to deal with that since I’m dealing with this heart issue.” (Participant 013, Patient)*

One caregiver agreed that different clinicians can have different interpretations of treatment for recurrent UTIs in particular:

*“She had one urologist that told her she would always have to deal with UTIs and she would just have to live with it, and another one that was like, “No, that’s ridiculous”…there’s been conflicting stories.“ (Participant 022, Caregiver)*

**Theme 3 (Opportunity and Motivation): Some patients and caregivers worry that clinicians do not take older adults’ concerns seriously.**

One caregiver felt that a monthly urinalysis would help pick up UTIs earlier before they might spread; the patient’s clinicians were not willing to deviate from clinical guidelines leaving the caregiver feeling helpless in advocating for her family member.

*“Well…I know what happens to her when she gets a UTI. It is bothersome to me that she’s [the clinician’s] not willing to do a monthly UA [urinalysis]…they’re not doing what I asked…It’s a little frustrating. Not much I can do about it.” (Participant 007, Caregiver)*

Many understood that clinicians wanted to follow guidelines about antibiotics, but worried that symptoms might present differently for older adults:

*“I understand the need to not over prescribe antibiotics. I really do get that…My feeling about it is that because someone’s older, and they’re not cognitively intact, that there’s less attention paid, that it’s easy to dismiss the concerns of that person, or not so much of that person but of their family. Yeah, I think that’s just generally true in treating older adults.” (Participant 024, Caregiver)*

Some even went on to describe feeling dismissed when bringing up UTI concerns:

*“I disagreed [with not treating]. I didn’t understand why you wouldn’t treat something that’s there [like bacteria in the urine]…I just felt like I didn’t agree with what was being said… I felt patronized.” (Participant 024, Caregiver)*

*“The first doctor, he gave up on me…He said, “You’ll probably always have the UTIs and you’ll also have the burning for the rest of your life and bleeding too”…I went home and cried.” (Participant 021, Patient)*

**Theme 4 (Opportunity and Motivation): Most participants stated that they trusted information from their clinicians over brochures or websites, but would like to review information to supplement clinical conversations.**

Caregivers and patients agreed that they would like to review information after clinical visits to better understand UTIs and their symptoms:

*“Well, I don’t know, maybe if I had some kind of…fact sheet, even just something that said a little more detail. That might be helpful…if the staff there would have said ‘Here, I’m gonna send you this link; it’s got some more information on UTIs.’” (Participant 004, Caregiver)*

*“If you could get from your doctor’s office…I think a lot of people in my generation still like the hands-on thing that you can take home and read.” (Participant 013, Patient)*

*“I’ve been reading on my cell phone…I read some information on the computer. I usually read whatever I can pick up at the doctor’s office for pamphlets or handouts and try to stay up on what’s going on with it.” (Participant 016, Patient)*

*“Having it to read [after a visit] would be helpful for me…Then I can go back to it and refer to it and read it again to refresh.” (Participant 021, Patient)*

A few participants cautioned that the brochure or website should be high quality in order to trust it:

*“[Some] pamphlets and brochures just tend to raise more questions for me…sometimes I feel like pamphlets kind of dumb things down.” (Participant 024, Caregiver)*

*“I’ve gone on the internet and read articles about the best way to treat…I’ll run that past-whatever I found past my doctor and make sure that she’s okay with it.” (Participant 001, Patient)*

**Theme 5 (Capability): Some participants understood that antibiotics should be taken cautiously because of potential for resistance at an individual level, but no one mentioned the public health significance of antibiotic resistance at the population level.**

*“Don’t take antibiotics unnecessarily because they…destroy good bacteria in your gut and elsewhere in your body, and then you can become resistant to them…you may develop a tolerance for a certain dose, and then you need more, and it’s an endless cycle.” (Participant 001, Patient)*

*“If you, on a regular basis, take these antibiotics when you don’t need them…it’s a red flag because the more you take them without needing it, when you do need it, they [bacteria] may not respond.”(Participant 015, Patient)*

*“One of the doctors told me not have them do anymore [take antibiotics] here because you get too many of ‘em…the antibiotics don’t work or something…You build up a resistance to antibiotics, so I shouldn’t be having them all the time here.” (Participant 021, Patient)*

**Theme 5a.** A minority of participants were relieved to learn that antibiotics were not always needed for bacteria in the urine without symptoms.

*“What I really liked hearing is that…you do not have to be on antibiotics…when that’s drummed into your head that for every cut and scrape and problem that you might have, you have to have an antibiotic, I really like hearing that you don’t…“I think maybe it’s outmoded our outdated that in the medical profession that there’s ‘a pill for every ill.’ I like the fact that that isn’t true.” (Participant 013, Patient)*

*“A lot of senior citizens…don’t like taking a lot of extra pills and that’ll be one less pill they have to take themselves or the caregiver has to monitor.” (Participant 020, Patient)*

**Theme 6: Despite recognizing the potential for antibiotic resistance if antibiotics are taken too often when they are not needed, most found the benefits of taking antibiotics for bacteria in the urine overall positive, especially because they worried about progression to worse symptoms.**

*“[I worry] that maybe that bacteria can continue to grow and eventually cause an infection—a worse infection, a more significant infection… Well, that other organisms may start to grow.” (Participant 007, Caregiver)*

*“I would think that if then your body is not functioning at the highest level to get rid of the bad bacteria on its own, that there could be some damage being done to organs.” (Participant 004, Caregiver)*

*“[if left untreated]…maybe that it would lead to something else that would be worse… because if you’re already fighting an infection, and your body can’t respond and heal it, then what? Your immune system is gonna be compromised.” (Participant 013, Patient)*

*“…so I have bacteria in my urine, I don’t have any symptoms right now, but…all of a sudden I’m doubled over in pain, maybe I could have eliminated that or prevented that if I had had a course of antibiotics for the bacteria that was there.” (Participant 024, Caregiver)*

## Discussion

These interviews with older adults with a history of UTIs and their caregivers identified several important themes that should be incorporated into future ASB and UTI interventions. Patients and caregivers reported confusion and distress about the diagnosis, treatment, and communication around ASB and UTIs. These findings represent opportunities to improve patient and family interactions about ASB and UTI management. To our knowledge, this is one of the first studies to incorporate patient and family perspectives on this important topic.

Although we found that older adults and caregivers generally understood common symptoms of UTIs, several struggled to evaluate nonspecific symptoms, such as confusion. This difficulty could result from variable information from experts: several participants felt that healthcare providers could give conflicting and sometimes incorrect information about ASB and UTIs. In particular, experts should better explain that ASB can be a benign condition. Perhaps clinicians could emphasize data showing that treating ASB can paradoxically increase the risk of a UTI in the future.^22,23^ Furthermore, clinician communication skills training might help increase the transparency of the care team decision-making process to address patients’ misunderstanding or fears about ASB.^24,25^

Beliefs about why antibiotics should be minimized for ASB varied by participant. Although some participants understand the broad relationship between antibiotic use and antibiotic resistance, they described this concern at the individual rather than societal level. In particular, they were most concerned about whether taking a course of antibiotics could decrease the effectiveness of antibiotics should they develop a bacterial infection in the future. Patient educational materials should emphasize these individual-level concerns over information about societal or global antibiotic resistance to increase the effectiveness of messaging.

One-on-one education from a healthcare provider was more trusted than passive educational materials, such as pamphlets, posters, or internet resources. However, participants wanted educational materials that would reinforce clinical counseling. This result is consistent with the literature that passive interventions such as pamphlets and posters provide only modest reductions in antibiotic prescribing for non-indicated conditions.^26^ Communication from a trusted healthcare provider could enhance the effectiveness of information presented in pamphlets, posters, or internet resources by describing the rationale and purpose of not treating conditions like ASB. This multifaceted information exchange could support patients’ and families’ understanding and trust in decisions about ASB management.

Clinician-level interventions to reduce the frequency of urinary culture testing when not indicated (e.g., diagnostic stewardship) could support appropriate antibiotics prescribing. Diagnostic stewardship could reduce the distress caused by patients or caregivers who may fixate on ridding the body of any bacteria. Interventions that prompt clinicians to obtain urine testing only in the presence of UTI symptoms work well^27^. However, any clinician-focused intervention should also include a patient and caregiver component. Our data clearly show that patients and caregivers may feel ignored if they are not provided a detailed rationale for avoiding urine testing or antibiotics for ASB.

Findings should be interpreted within the context of study limitations. Our participants were mostly women from the Midwestern region of the US and included few members of minoritized populations. Furthermore, our population generally included highly educated individuals. These findings were likely an artifact of our recruitment approach, which focused on patients reaching out to us through newsletters and senior living facilities. Although we comfortably reached thematic saturation, greater diversity of participants might have captured new themes. We also focused on older patients and their caregivers as these individuals are more likely to have ASB or side effects from antibiotics; results may be less generalizable to other populations such as pregnant women or select cancer patients where ASB should be treated.

In summary, these data can support multi-level interventions to improve communication about urinary symptoms, information about ASB and UTIs, and decision support for urinary testing and treatment. Tools, brochures, and electronic health system interventions could reduce communication barriers and improve transparency in decision-making. The longer-term goal of this line of work is to develop and implement such tools, responding to patient, caregiver, and clinician needs and preferences about evidence-based ASB and UTI management.

## Supporting information

Durkin et al. supplementary material Durkin et al. supplementary material

## Supplementary material

For supplementary material accompanying this paper visit http://doi.org/10.1017/ash.2023.498. click here to view supplementary material

## Research transparency

De-identified data in the form of written transcripts can be made available upon reasonable request.
