# Supplementary material for: Older adults’ and caregivers’ perceptions about urinary tract infection and asymptomatic bacteriuria guidelines: a qualitative exploration
Source: Antimicrob Steward Healthc Epidemiol. 2023 Dec 4;3(1):e224. doi: 10.1017/ash.2023.498 (PMC10753467; doi:10.1017/ash.2023.498)
Supplement: Durkin et al. supplementary material [file S2732494X23004989sup001.docx]

**Stakeholders’ perceptions of consensus guidelines about antibiotics for asymptomatic bacteriuria.**

**Patient/Caregiver Interview Guide**

**Introduction:**

Thank you for meeting with us today. We really appreciate your time and insights. As a reminder, the information you provide is kept in strict confidence. The transcript that results from today’s interview will not identify you in any way. There are no right or wrong answers to any of today’s questions, and if you are not comfortable answering a question, you don’t have to answer. We’re interested in hearing your personal experiences, thoughts and feelings.

Before we begin, we’d like give you some more information about this project. We’ll be discussing treatment for bacteria in the urine. Sometimes the bacteria in the urine causes symptoms and needs to be treated. This is often called a urinary tract infection, or a UTI.

Other times, the bacteria in the urine is found by testing for it, but it is not causing any symptoms and might not need treatment.

We want to know what you know about UTIs and their symptoms, how you have been treated in the past for UTIs or bacteria in the urine, and how you talk to your health care team about symptoms.

**Overview questions**

- When was the last time you had a UTI?
  - If a caregiver: When was the last time they had an UTI?
- What were your symptoms, if you had any?

Probe: odor, pain, frequent urination, frequent urge to urinate, getting up at night to urinate

- What treatment did you receive for your UTI, if any?
- Could you please describe the process of how your doctor diagnosed you with the UTI
- Has a doctor or nurse ever told you that you had bacteria in the urine, but you did not need treatment?

**Capability**

- Are you familiar with the guidelines for treatment of bacteria in the urine, when someone does not have symptoms? If so, please explain

(*show guidelines to all participants after they explain what they know, to get on the same page*)

- What are some things you have been told about UTI's?
- Did you find the information that you were told misleading or confusing?

Probe: Where did you hear this misleading or confusing information? (If no skip to motivation)

- How did this information impact your treatment?

**Motivation**

- What UTI symptoms worry you?
- What do you think are risk factors for getting an UTI?
- Why do you think UTI occur frequently?
- How often do you think UTI can occur?
- What are some benefits of not treating bacteria if you are symptom free?
- What are some drawbacks of not treating bacteria if you are symptom free?

**Opportunity**

- Who helps make your decision with you about UTI treatment?

Probe: doctor, nurse, family, friends, caregivers)?

- Have you used any resources that helped you understand UTIs and their symptoms?
- What are some resources that would be helpful to better understand UTIs and their symptoms?
- What could make understanding this information challenging?

Before we end, is there anything else you’d like to tell us about bacteria in the urine or treatment for it?
